# Determinants of Emotional Intimate Partner Violence against Women and Girls with Children in Mexican Households: An Ecological Framework

**DOI:** 10.1177/08862605211072179

**Published:** 2022-02-08

**Authors:** Juan Armando Torres Munguía, Inmaculada Martínez-Zarzoso

**Affiliations:** 1 9375 Georg-August-Universität Göttingen, Gottingen, Germany

## Abstract

The purpose of this paper is to identify the risk factors for emotional intimate partner violence (IPV) against women and girls with children in Mexico from an ecological perspective. To that end, we generate a dataset with 35,004 observations and 42 covariates, to which we apply an additive probit model estimated with a boosting algorithm to overcome high-dimensionality and simultaneously perform variable selection and model choice. The dataset integrates 10 information sources, allowing us to properly characterize the four levels of the ecological approach, which is the first contribution of this paper. In addition, there are three key contributions. First, we identify a number of factors significantly linked to emotional IPV against women with children: age, age at sexual initiation, age at marriage (or cohabitation), autonomy regarding professional issues, social support networks, division of housework, the community’s Gini index, women’s economic participation in the municipality, and the prevalence of crime against males in the region. Second, we discuss some risk factors whose effects have not been examined or have been underexplored for Mexico; these include women’s decision-making autonomy, social support networks, distribution of housework, the community’s economic inequality, and criminality. Third, we identify specific risk subgroups that are generally overlooked: women who had their first sexual intercourse during childhood and women who got married (or moved in together with a partner) late in life. The main results suggest that strategies aiming to promote women’s social and economic empowerment and reduce criminality should also incorporate a gender component regarding emotional violence against women with children in the context of intimate relationships.

## Introduction

According to the 2016 National Survey on the Dynamics of Household Relationships (ENDIREH), the most prevalent act of intimate partner violence (IPV) against women in Mexico is emotional abuse, affecting approximately 40.1% (17.4 million) of all ever-partnered women aged 15 years or over (INEGI, 2016b).

The impact of emotional IPV is severe. Half of the victims experienced stress, depression, insomnia, and loss of appetite, and about 8.6% (1.5 million) of them have thought about killing themselves or have already attempted suicide (INEGI, 2016b). IPV also affects other family members, especially the children (Sturge-Apple et al., 2012), placing women with children at a particular risk regarding IPV (Peek-Asa et al., 2017).

Given the above-mentioned consequences, gaining a better understanding of what drives the risk of emotional IPV victimization is of paramount importance. One of the most widely used approaches to study the multifaceted nature of violence, including IPV, is the ecological model. According to this approach, violence can be explained as a result of the interaction and convergence of multiple factors at four interrelated levels: individual, relationship, community, and society (WHO, 2012).

For the case of Mexico, findings from research using the ecological model suggest that being young (Castro et al., 2006; Villarreal, 2007), having a low education level (Avila-Burgos et al., 2009; Jaen Cortés et al., 2015; Rivera-Rivera et al., 2004), from a low socioeconomic status (Castro et al., 2006; Castro & Casique, 2008) and being in a relationship with a young man (Casique & Castro, 2014) who abuses alcohol/drugs (Mojarro-Iñiguez et al., 2014), displays controlling behavior (Frías, 2017), has a history of violence victimization/perpetration (López Rosales et al., 2013), or who is unemployed (Valdez-Santiago et al., 2013), are risk factors for women’s IPV victimization.

Despite the importance of these findings, there are still some theoretical factors in the ecological approach, whose relevance is generally acknowledged in international studies (UNiTE Working Group, 2019; WHO, 2012), but their effects have not been examined in-depth—or not at all—for Mexico. Three of them belong to the individual level: young age at first childbirth, unwanted sexual initiation at an early age, and lack of a pro-gender equality attitude. Four additional under-studied factors belong to the relationship level: getting married young and not by choice, a lack of decision-making power, unequal distribution of housework to women’s detriment, and a lack of social support networks. At the community level, there are two factors: risks of women living in communities with unequal income distribution and a low level of women’s participation in the public sphere. Similarly, at the societal level, the effects of living in a society with low quality of government, high corruption levels, and high rates of criminal activity on IPV victimization have also not yet been examined for Mexico. The analysis of these factors is vital since identifying their effects could help policy-makers not only to design specific strategies to protect current victims but to develop early interventions targeted at high-risk communities and regions, and the most vulnerable population groups, in order to prevent future aggressions (Heise, 2011).

Intending to contribute to the discussion on the emotional IPV risk factors in Mexico, emphasizing the above-mentioned under-studied factors, we examine how and to what extent a set of theoretical factors are linked to women’s probability of victimization. To that end, we apply a probit model to data on the victimization experiences of 35,004 women and girls with children in Mexico. The population studied includes women and girls aged 15 years and over, which allows us to capture how the risks vary over their lifetime, from adolescence to old age, while controlling for the rest of the factors in the model. Our data contain more than 40 potential variables at the four levels of the ecological model, taken from 10 official data sources. The creation of this dataset enables us to overcome two shortcomings in IPV studies identified by Krug et al. (2002) in the *World report on violence and health*: the inclusion of a limited number of potential risk factors and the lack of characterization of the community and society where violence occurs.

The rest of this paper is organized as follows. Section 2 briefly introduces our method, including the theoretical framework, data sources, variables, and statistical analysis. Section 3 details the findings, and section 4 provides some potential explanations for the results. Finally, section 5 presents the conclusions and final remarks.

## Literature Review

### An Ecological Model for Understanding Intimate Partner Violence

According to the ecological model, IPV is grounded in a combination of factors operating at four different levels: individual, relationship, community, and societal. It is critical to note that each of these factors interacts not only with the rest of the factors within their corresponding level but also with those from the other levels. These interactions play a crucial role since no single factor can explain IPV, but rather a myriad of them shape the women’s victimization risks (WHO, 2012). To briefly discuss the factors identified as relevant across studies from different countries most consistently, we present some findings at each level of the ecological model to analyze the Mexican case.

### Individual-Level Factors

At the individual level, there is evidence suggesting that IPV is more prevalent among women and girls from minority groups, with a low level of economic empowerment, who had their first childbirth at an early age, their first sexual intercourse at a young age and/or against their wishes, and who lack a pro-gender equality attitude (WHO, 2012). Findings supporting the relevance of these variables can be found in the study by Oduro et al. (2015) for the cases of Ecuador and Ghana, by Stöckl et al. (2014) using data from a multi-country study by Cameron and Tedds (2021) with data from Canada, and by Caetano et al. (2005) with data from the United States.

Women’s age has also been found to be significant for IPV, yet findings suggest that this association has a context-specific effect. Using data from the United States, Walton-Moss et al. (2005) found that larger risks are observed among young women. By contrast, Wilson (2019), using data from 36 countries, concluded that particularly for emotional and physical IPV, the association with age is described by an inverted U-shaped curve. Findings also indicate that the intersection of women’s age with other demographic characteristics alters the IPV risks. For instance, Heidinger (2021) found that the gap in victimization risks between indigenous and non-indigenous Canadian women varies over their lifetime, reaching a maximum between 25 and 44 years old. A similar pattern is described for the interaction of age with education level in the report by Oficina de Violencia Doméstica (2021) with data from Argentina.

### Relationship-Level Factors

The second level of the ecological model captures the features of the woman’s closest social circles: her intimate relationship and her relationship with peers and family.

Regarding the intimate relationship, some studies have found a number of the partner’s characteristics correlated with IPV: young age, low education levels, frequent alcohol consumption, and living in economic disadvantage (National Center for Injury Prevention and Control, 2020; WHO, 2012). These results are confirmed by Stöckl et al. (2021) with data from Sub-Saharan Africa, Caetano et al. (2001) for the United States, Stöckl et al. (2012) for the German case, and Ahmadabadi et al. (2020) for Australia.

Nevertheless, the above-mentioned features are not risk factors per se but instead refer to the woman’s situation relative to her partner. For instance, Rapp et al. (2012) concluded that lower IPV risks are observed among couples with the same education level in India and Bangladesh. In the same vein, results reported in Abramsky et al. (2019) for the case of Tanzania, and in Reichel (2017) with data from the European Union countries, lend support to the idea that discrepancies between the woman’s economic status and that of her partner lead to higher IPV risks. Similarly, Chaurasia et al. (2021) found that a large age gap exacerbates the likelihood of experiencing IPV in India.

Moreover, women’s autonomy has been found to be negatively correlated with IPV in Pakistan (Mavisakalyan & Rammohan, 2021) and Turkey (Yilmaz, 2018).

Regarding the woman’s relationship with her peers and family, research shows that an unequal distribution of housework, overcrowding, and inadequate social support networks are factors that increase IPV. Among others, such findings have been reported by Wright (2015) with data from Chicago, Nguyen et al. (2018) for pregnant women in Vietnam, and Plazaola-Castaño et al. (2008) in three autonomous communities in Spain.

### Community-Level Factors

At the community level, findings indicate that women living in urban settlements, in communities with high crime incidence, high concentration of immigrants, unfavorable socioeconomic circumstances, and/or gender-inequitable conditions are at greater risk of IPV (UNiTE Working Group, 2019; WHO, 2012). Some papers coming to these conclusions are those by Dias et al. (2020), studying six European cities, Lauritsen and Schaum (2004) and Voith et al. (2021) with data from the United States, and Ackerson and Subramanian (2008) for India.

### Societal-Level Factors

At the fourth level of the ecological model, as found by Gillanders and van der Werff (2020) for the case of African countries with data from the Afrobarometer, Gashaw et al. (2018) for Ethiopia, and González and Rodríguez-Planas (2020) using survey data from 28 European countries, the most consistent risk factors at the societal level include low quality of government, high crime incidence, social instability, and high prevalence of sexist norms and beliefs.

### Previous Research Analyzing Intimate Partner Violence in Mexico

Even though studies for Mexico have tended to apply the ecological model, they have almost exclusively analyzed the association of individual- and relationship-level factors with IPV.

Regarding the individual-level factors, Castro et al. (2006) and Villarreal (2007) used data from the 2003 ENDIREH to show that younger women are potentially more at risk of IPV. In addition to age, Jaen Cortés et al. (2015) and Rivera-Rivera et al. (2004) found that women’s education level is negatively associated with IPV victimization. Moreover, IPV is also more prevalent in women from low socioeconomic backgrounds (Castro et al., 2006; Castro & Casique, 2008).

Regarding the relationship-level factors, Casique and Castro (2014) and Castro et al. (2006) found that women with a young partner are more likely to suffer from IPV. Moreover, Rivera-Rivera et al. (2004), Esquivel-Santoveña et al. (2020), and Terrazas-Carrillo and McWhirter (2015) showed that other key IPV risk factors are the partner’s heavy drinking and controlling behavior. By examining data from Monterrey in Mexico, López Rosales et al. (2013) found that higher risks are expected in women whose partners have a history of violence perpetration and/or victimization. The partner’s socioeconomic disadvantages (low education level or unemployment) are also expected to be risk factors for IPV (Alvarado-Zaldívar et al., 1998; Avila-Burgos et al., 2009; Valdez-Santiago et al., 2013).

The community level remains largely under-studied for Mexico. Only Castro and Casique (2009) distinguished between IPV risks in urban and rural communities, while Valdez-Santiago et al. (2013) analyzed data from some indigenous regions in Mexico to study the prevalence and severity of IPV and introduced covariates such as community type and poverty level in the municipality.

Concerning the societal level, only a handful of papers have considered single factors at this level of analysis for Mexico. García-Ramos (2021), analyzing state-level data over time, found that divorce laws significantly affect IPV in the long term, while Sterling (2018) argued that a sexist culture is strongly linked to a high risk of IPV in Mexico.

## Method

### Data Sources

After identifying a set of theoretical factors at the four levels of the ecological model, we map the official data sources containing this information for Mexico. Our main source is the 2016 ENDIREH, from which we obtain data at the individual and relationship levels. To characterize the community and societal levels, we use the unique identifier of the respondent’s residence from the ENDIREH to match their individual responses with the corresponding information on the municipality and state from nine other official sources. This integration process is briefly presented below, and a more detailed description of it can be found in Supplemental Material A.

### An Overview of the ENDIREH

The ENDIREH is a nationally representative household survey conducted by Mexico’s National Institute of Statistics and Geography (INEGI). This survey aims to produce information on the violence experienced by women and girls aged 15 years and over in Mexico. The survey explores four types of violent acts, namely physical, sexual, economic, and emotional, which occur in the contexts of the community, workplace, and school environments, in the family, and within intimate relationships. For this research, we only use information from the questionnaire referring to heterosexual married or cohabitation women.

### Other Sources of Data

As described in Supplemental Material A, to characterize the community and societal levels, we identify in the ENDIREH the municipality and state where the respondent lives. Then, we merge the information about the individual and relationship levels from the ENDIREH with the estimations from the official poverty data generated by the National Council for the Evaluation of Social Development Policy (CONEVAL), marginalization data from the National Population Council (CONAPO), the municipal geographical information and homicide records collected by the INEGI, the human development index produced by the United Nations Development Program (UNDP), information from the 2015 Intercensal Population Survey, the 2016 National Survey on Victimization and Perception of Public Safety (ENVIPE), the 2015 National Census of Municipal and Delegation Governments (CNGMD), and from the 2015 National Survey of Quality and Governmental Impact (ENCIG). More details on these sources can be found in CONAPO (2016), CONEVAL (2020), INEGI (2015a, 2015b, 2015c, 2016a, 2016b), and UNDP (2019). These datasets are freely available at www.inegi.org.mx, www.coneval.org.mx, www.conapo.gob.mx, and www.mx.undp.org.

## Variable Description

### Dependent Variable

Our dependent variable takes the value of one if a woman has suffered from emotional IPV and 0 otherwise. Information on victimization is produced via self-reported responses to a question in the ENDIREH asking about the occurrence of 15 emotional violence acts suffered in the context of their current or previous relationship in the preceding 12 months, *that is.,* between October 2015 and October 2016 (see Table 1 for the list of acts and behaviors included). Possible responses to this question are “many times,” “sometimes,” “once,” and “never”. Given that the frequency associated with “many times” and “sometimes” is not precisely defined but rather is left to the respondent’s own judgment, we decide to generate a binomial variable by dichotomizing the answers into “yes” or “no” where the three first responses are considered as “yes.” This allows us to focus specifically on the probability of experiencing emotional IPV.

**Table 1.** Acts of Emotional IPV Measured by the 2016 ENDIREH.

**Type of act**	**Behaviors included**
Social isolation	· Forbidding the woman to leave the house, locking her up, or stopping her from having visits.
	· Turning children or relatives against the woman.
Threats	· Threatening the woman about abandoning her, to harm her, to take the children, or to kick her out the house.
	· Threatening the woman with a weapon.
	· Threatening the woman to kill her, to kill himself or to kill the children.
Humiliation	· Humiliating her, degrading her, comparing her with other women or calling her ugly.
	· Blaming her on cheating on him.
Indifference	· Ignoring her, embarrassing her, not taking her into account or not giving her affection.
	· Stop talking to the woman.
Intimidation and stalking	· Making her feel scare.
	· Stalking her, spying her, following her around, showing up suddenly in places.
	· Calling or texting the woman repeatedly to know her location, if she is with someone and what she is doing.
	· Destroying, throwing, or hiding personal or household property.
	· Monitoring woman's mails or cellphone and demanding passwords.
	· Reproaching and getting angry with the female because household chores are not done in the way the male partner wants, because food is not done or because he considers she does not fulfill her obligations.

Source: Own elaboration based on INEGI (2016b)

### Independent Variables

Following the ecological approach and previous studies, after selecting the data sources, we identify the available theoretical factors proposed in the literature review at the individual, relationship, community, and societal levels. The full list of potential explanatory variables included in this study is listed in Table 2.

**Table 2.** List of Covariates Included in the Model by Level of the Ecological Model.

**Level**	**Variable (type)**	**Alternative effects in the model**	**Source**
***Individual***	***Demographic factors:***		
	· Age of the woman in years (continuous)	Linear and nonlinear	ENDIREH
	· Indigenous origin of the woman (categorical: “yes,” “no”)	Linear	ENDIREH
	· Formal education level of the woman (categorical: “low”, “medium”, “high”)	Linear	ENDIREH
	· Age of the woman by indigenous origin (continuous)	Interaction	ENDIREH
	· Age of the woman by education level (continuous)	Interaction	ENDIREH
	***Economic factors:***		
	· Woman’s reported monthly earned income, in Mexican Pesos (continuous)	Linear and nonlinear	ENDIREH
	***Gender-related factors:***		
	· Age of the woman at first childbirth (continuous)	Linear and nonlinear	ENDIREH
	· Age of the woman by age at first childbirth (continuous)	Interaction	ENDIREH
	· Age of the woman at her first sexual intercourse (continuous)	Linear and nonlinear	ENDIREH
	· Consent to first sexual intercourse (categorical: “yes”, “no”)	Linear	ENDIREH
	· Age of the woman at her first sexual intercourse by condition of consent (continuous)	Interaction	ENDIREH
	· Age of the woman by age at her first sexual intercourse (continuous)	Interaction	ENDIREH
	· Pro-gender equality attitude (categorical: "low", “medium”, “high”)	Linear	ENDIREH
***Relationship***	***Demographic factors:***		
	· Age of the husband or partner in years (continuous)	Linear and nonlinear	ENDIREH
	***Economic factors:***		
	· Husband’s or partner’s reported monthly earned income, in Mexican Pesos (continuous)	Linear and nonlinear	ENDIREH
	***Gender-related factors:***		
	· Age of the woman at marriage or at cohabitation (continuous)	Linear and nonlinear	ENDIREH
	· Consent to marriage or cohabitation (categorical: “yes,” “no”)	Linear	ENDIREH
	· Age of the woman at marriage or at cohabitation by condition of consent (continuous)	Interaction	ENDIREH
	· Age of the woman by age at marriage or at cohabitation (continuous)	Interaction	ENDIREH
	· Age of the woman by age of the husband or partner (continuous)	Interaction	ENDIREH
	· Woman's level of autonomy within the relationship to make decisions about her sexual life (categorical: “low,” “medium,” “high”)	Linear	ENDIREH
	· Woman's level of autonomy within the relationship to make decisions about her professional life and use of economic resources (categorical: “low,” “medium,” “high”)	Linear	ENDIREH
	· Woman's level of autonomy within the relationship to make decisions about her participation in social and political activities (categorical: “low,” “medium,” “high”)	Linear	ENDIREH
	· Woman’s reported monthly earned income by reported husband’s or partner’s reported monthly earned income, in Mexican Pesos (continuous)	Interaction	ENDIREH
	***Household characteristics:***		
	· Average number of household members per room in the dwelling (continuous)	Linear and nonlinear	ENDIREH
	***Women's role and participation in the household:***		
	· Division of housework among household members (categorical: “only women,” “both,” “only men”)	Linear	ENDIREH
	***Close-community characteristics (neighborhood, friends):***		
	· Women's perception about having support from social networks (categorical: “low,” “medium,” “high”)	Linear	ENDIREH
	· Level of social interaction reported by the woman (categorical: “low,” “medium,” “high”)	Linear	ENDIREH
***Community***	***Demographic characteristics of the Municipality:***		
	· Male share of recent migrant population (continuous)	Linear and nonlinear	Intercensal Population Survey
	· Level of social marginalization (categorical: “very low,” “low,” “medium,” “high,” “very high”)	Linear	CONAPO
	· Type of community (categorical: “rural,” “low urban,” “medium urban,” “high urban”)	Linear	CONAPO
	***Economic characteristics of the Municipality:***		
	· Human development index in 2015 (continuous)	Linear and nonlinear	UNDP
	· Gini index 2015 (continuous)	Linear and nonlinear	CONEVAL
	· Economically active men population in 2015 (continuous)	Linear and nonlinear	Intercensal Population Survey
	***Public security characteristics of the Municipality:***		
	· Total homicide rate per 100,000 inhabitants in 2015 in the Municipality (continuous)	Linear and nonlinear	Homicide records
	· Men homicide rate per 100,000 men in 2015 in the Municipality (continuous)	Linear and nonlinear	Homicide records
	***Government characteristics of the Municipality:***		
	· Municipal functional capacities index in 2016 (continuous)	Linear and nonlinear	UNDP
	***Women's role and participation in the Municipality***		
	· Share of senior positions in the Municipal Public Administration held by women in 2015 (continuous)	Linear and nonlinear	CNGMD
	· Economically active women population in 2015 (continuous)	Linear and nonlinear	Intercensal Population Survey
	· Women homicide rate per 100,000 women in 2015 in the Municipality (continuous)	Linear and nonlinear	Homicide records
	· Female share of recent migrant population (continuous)	Linear and nonlinear	Intercensal Population Survey
	· Share of the population living in women-headed households in 2015 (continuous)	Linear and nonlinear	Intercensal Population Survey
	***Geographic information of the Municipality:***		
	· Municipality of residence	Random	ENDIREH
	· Centroid coordinates: longitude, latitude	Spatial	Geographic information
***Societal***	***Government characteristics of the State:***		
	· Share of the population who considered corruption a common or very common problem in their region in 2015 (continuous)	Linear and nonlinear	ENCIG
	· Share of the population satisfied with the basic public services in their region in 2015 (continuous)	Linear and nonlinear	ENCIG
	***Public security characteristics of the State:***		
	· Share of common crimes against men not reported to or not registered by the authorities in 2015 (continuous)	Linear and nonlinear	ENVIPE
	· Prevalence rate of common crimes against men per 100,000 men in 2015 (continuous)	Linear and nonlinear	ENVIPE
	***Women's role and situation in the State:***		
	· Share of common crimes against women not reported to or not registered by the authorities in 2015 (continuous)	Linear and nonlinear	ENVIPE
	· Prevalence rate of common crimes against women per 100,000 women in 2015 (continuous)	Linear and nonlinear	ENVIPE
	***Geographic information of the State:***		
	· State of residence	Random	ENDIREH

As shown in the third column of Table 2, three alternative effects are considered in the model. First, purely linear effects are introduced for categorical covariates. Second, for continuous variables, instead of imposing a priori a particular linear form on them, we test both linear and nonlinear effects. As discussed in the literature review, there is empirical evidence suggesting the existence of nonlinearities in some factors, such as women’s age (Wilson, 2019). Finally, the introduction of interaction effects is justified for three reasons. First, the literature review indicates that some categorical variables at the individual level, such as indigenous origin and education level, alter the effect of women’s age on IPV (Doméstica, 2021; Heidinger, 2021). The same occurs with the categorical variable condition of consent with age at sexual initiation and marriage (or cohabitation). The second reason is to capture relative inequalities in age and income between the woman and her partner, as studied by Rapp et al. (2012), Chaurasia et al. (2021), and Reichel (2017). The third reason for including the interactions is the definition of the factors, which should be considered in the modeling design. This is the case of the interaction between the woman’s age and age at first childbirth. Age at first childbirth depends on the value of a woman’s age. Moreover, the effect of having had the first child at, say, 16 years old would be different for an 18-year-old girl than for a 50-year-old woman. Something similar happens with the interaction of a woman’s age with age at marriage (or cohabitation).

Due to the hierarchical data structure, in which individual observations are connected to the information for the municipalities, and these, in turn to the state information, we introduce random effects. To explore whether IPV follows a particular spatial pattern, as found by Ojifinni et al. (2021), we also include the municipal centroid coordinates.

After merging the data sources and identifying the available relevant variables, we checked for plausibility, detected outliers, and removed missing cases to prepare the data for the analysis. A description of this data cleaning process can be found in Supplemental Material B. The final dataset is composed of 35,004 observations, which correspond to women who, at the time of being surveyed, were aged 15 or over, were married or cohabitating with a male partner, and had had at least one child. Summary statistics of these data can be found in Supplemental Material C.

### Statistical Analysis

In this paper, we apply a probit regression model adjusting for the sampling design and survey weights. The probit methodological alternative allows us to deal with the dichotomous nature of our dependent variable, whose binary outcome indicates whether the woman surveyed has suffered from emotional IPV during the reference period. In order to introduce linear, nonlinear, interaction, random, and spatial effects for the covariates in the model, we propose an additive structure as used by Friedman et al. (2000) and Hastie and Tibshirani (1999). On this basis, in the probit approach with the additive structure, the inverse standard normal distribution of the women’s likelihood of emotional IPV victimization is modeled as an additive combination of their risk factors (Friedman et al., 2000). Details on the modeling design can be found in Supplemental Material D.

Given the high-dimensionality and complexity of the model, we implement a three-step methodology. First, we perform the estimation, variable selection, and model choice via the boosting algorithm (Friedman, 2001; Hofner et al., 2014; Hothorn et al., 2020). Then, we apply complementary pairs stability selection with *per family* error rate control to avoid falsely selecting covariates (Meinshausen & Bühlmann, 2010; Shah & Samworth, 2013). Finally, we calculate the corresponding confidence intervals of the selected relevant variables (Hofner et al., 2014). Details on the estimation strategy are provided in Supplemental Material E.

## Results

Only nine effects are selected as significantly associated with emotional IPV victimization once the model is optimized from the 42 theoretical covariates included in the entire model. These results are summarized in Table 3 and discussed in the following paragraphs according to their corresponding level of the ecological model.

**Table 3.** Selected Variables Associated with Emotional IPV Victimization.

**Level**	**Variable**	**Categories**	**Coefficient [95% CI])**
Individual	Women's age		**Linear, slope: -0.003 (Figure 1.a)**
	Women's age at first sexual intercourse by condition of consent to first sexual intercourse	no	**Linear, slope: -0.012 (Figure 1.b)**
		yes	**Linear, slope: -0.018 (Figure 1.b)**
Relationship	Women's age at marriage with current husband or at moving in together with current partner by condition of consent to marriage or moving in	no	
		yes	**Linear, slope: 0.003 (Figure 2)**
	Women's autonomy about her professional life and use of economic resources	low*	
		medium	**- 0.1 [-0.129, -0.063]**
		high	
	Social networks	low*	
		medium	**0.079 [0.062, 0.097]**
		high	
	Division of housework among household members	only women	
		both	
		only males	**-0.07 [-0.086, -0.054]**
Community	Gini index		**Nonlinear, inverted u-shape (Figure 3.a)**
	Economically active women population		**Linear, slope: 0.002 (Figure 3.b)**
Society	Prevalence of common crimes against men		**Linear, slope: 0.000003 (Figure 4)**

### Individual-Level Risk Factors

**Figure 1.**
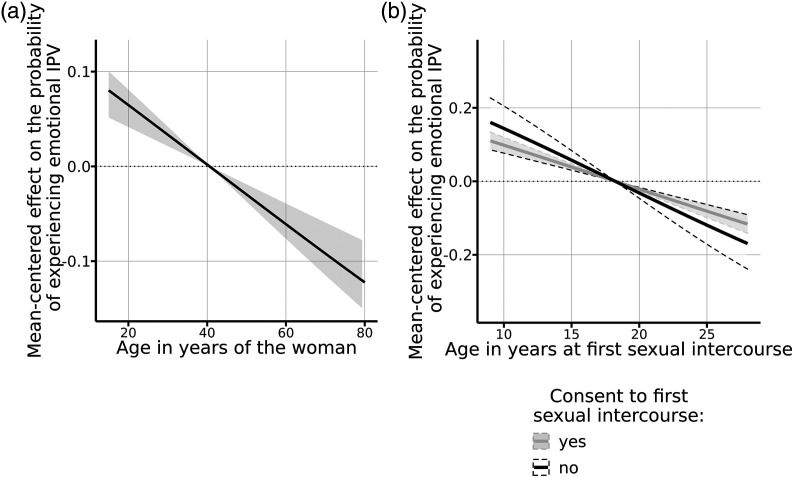
Effects of selected continuous emotional IPV covariates at the individual level. a) Emotional IPV risk and women’s age, b) Emotional IPV risk and women’s age at first sexual intercourse by consent. Note. IPV=Intimate Partner Violence.^1^

Two effects are found to be significant for emotional IPV at the individual level. First, regarding the effect of age on victimization, we find a linear decreasing relationship, suggesting that young women are at the most risk of victimization (Figure 1a). Specifically, the risk of emotional victimization for girls around 15 years old is approximately eight percentage points higher than for women aged 40 and about 20 points higher than for women aged 80. Women’s age at first sexual intercourse is also relevant for emotional IPV (see Figure 1.b). Results indicate that women who had their sexual initiation at an early age are generally at higher risk of suffering emotional IPV. This effect does not differ between women who consented to their first sexual experience and those who did not.

### Relationship-Level Risk Factors

**Figure 2.**
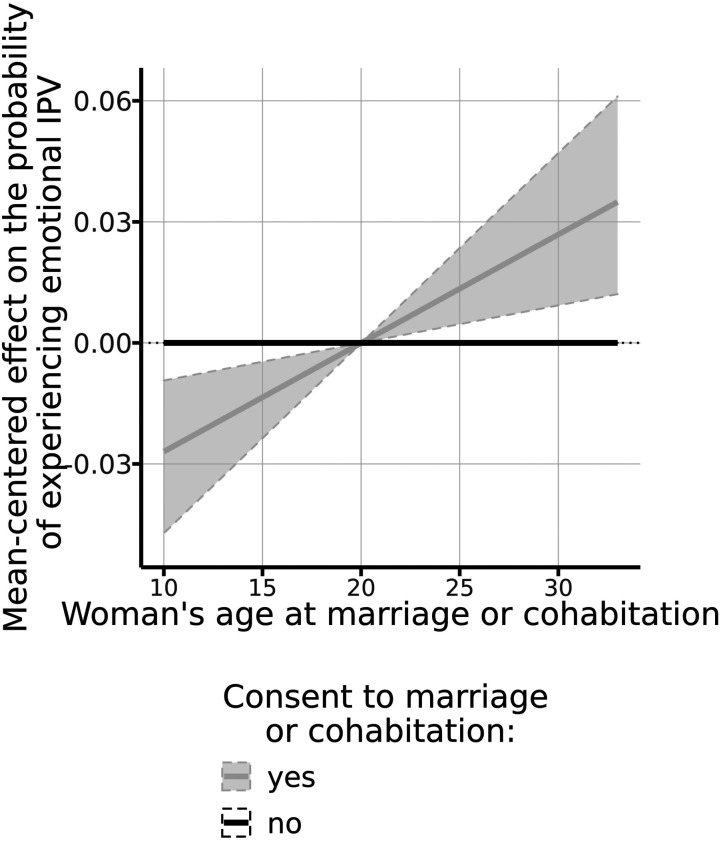
Effects of selected continuous emotional intimate partner violence covariates at the relationship level.^1^

At the relationship level, women’s age at marriage or cohabitation is positively associated with the likelihood of experiencing emotional IPV only for those who consent to it. Specifically, this association is represented by a line increasing at a constant rate of 0.3 percentage points per year of age (Figure 2).

A woman’s decision-making autonomy about her professional life is also a relevant factor for emotional IPV victimization (see Table 3). Results indicate that compared to women with poor decision-making power, women with a medium level of autonomy are at less risk of emotional IPV victimization. No significant differences are observed between women with low and high autonomy levels. We also find that women who have a medium level of social support networks experience, on average, a higher risk of emotional IPV than those with low and high perceived social connectedness (about eight percentage points more). Furthermore, results indicate that partnered women in families in which the housework is done by the male members exhibit a risk of emotional IPV that is around seven percentage points lower than that of women in households with a different distribution of housework (see Table 3).

### Community-Level Risk Factors

**Figure 3.**
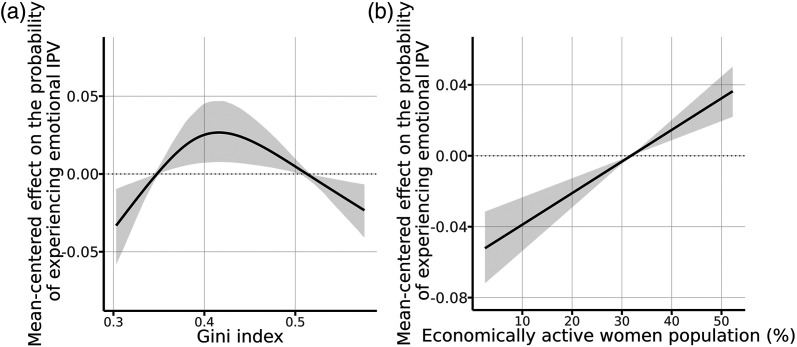
Effects of selected continuous emotional IPV covariates at the community level. a) Emotional IPV victimization risk and community’s Gini index, b) Emotional IPV victimization risk and community’s economically active female population. Note. IPV=Intimate Partner Violence.^1^

Concerning the community level, we find that the association between economic inequality, measured by the Gini index of the community, and the likelihood of a woman experiencing emotional IPV follows an inverted U-shaped curve (see Figure 3a). Moreover, the participation of women in the community’s economic activity is positively associated with IPV risks, as can be observed in Figure 3b.

### Societal-Level Risk Factors

**Figure 4.**
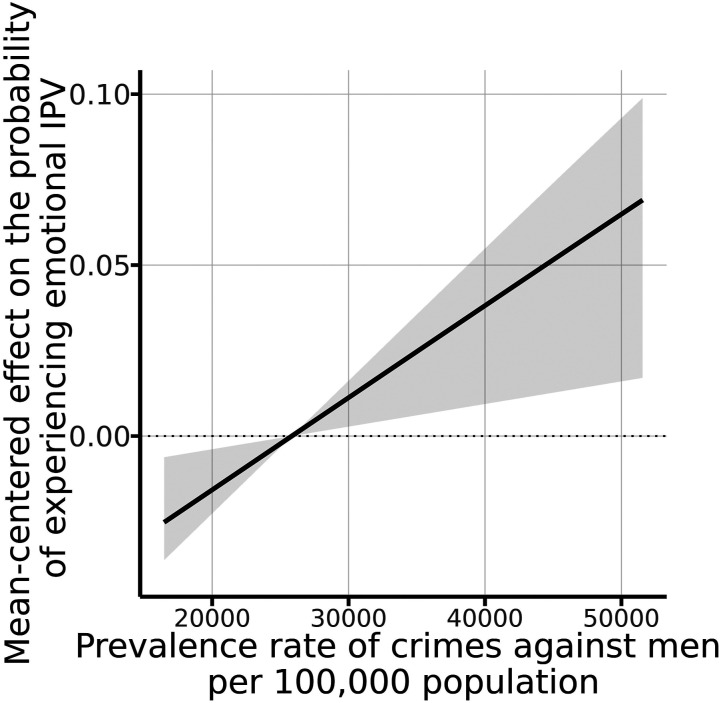
Effects of selected continuous emotional intimate partner violence covariates at the societal level.^1^

At the societal level, we find that the prevalence of common crimes against men in the region is positively associated with the likelihood of women and girls experiencing emotional IPV (see Figure 4). Women and girls living in regions with a prevalence rate of around 50,000 male victims per 100,000 men show a risk of emotional IPV approximately six percentage points higher than those living in regions with a rate around the national mean of approximately 30,000 male victims per 100,000 male inhabitants.

## Discussion

The significant factors presented in the previous section imply relationships between the selected covariates and the likelihood of emotional IPV victimization. Even though these relationships do not necessarily imply causality, they provide evidence about important aspects of emotional IPV in Mexico. This section discusses some possible explanations drawn from studies and theories presented in the literature review.

As pointed out by Walton-Moss et al. (2005), WHO (2012), and UNiTE Working Group (2019), the reasons underlying the age-victimization decreasing relationship might be related to the development of empowerment strategies and life skills throughout a woman’s life.

Regarding the negative correlation between the emotional IPV risk and age at first sexual activity, this result aligns with those of other international studies (Stöckl et al., 2014) and lends support to the argument that experiences during childhood and adolescence have a major long-run impact on individuals’ physical, mental, and social health. In particular, an early sexual experience is associated with many negative outcomes (Olesen et al., 2012).

Although our results regarding the positive linkage between women’s age at marriage (or at cohabitation) and her probability of experiencing emotional IPV contradict previous findings (WHO, 2012), there are two potential interpretations. On the one hand, it is generally expected that women who marry at a late age have greater economic power and better social opportunities (Field et al., 2016), and this could be prompting their partners to inflict IPV in an attempt to control their resources (Bloch & Rao, 2002). On the other hand, women who marry at a late age might be “tolerating” emotional IPV to avoid being unmarried and the subsequent lingering social stigma existing in Mexico (Cuevas Hernández, 2010; Médor, 2013) and because of concern for their children (UNiTE Working Group, 2019).

This result partially agrees with existing studies regarding the significant effect of women’s decision-making autonomy (Mavisakalyan & Rammohan, 2021). It could indicate that when a woman has a low level of autonomy regarding her professional life and use of resources, her partner exercises dominance and control over her through emotional violence. As a woman’s autonomy increases to a medium level, emotional IPV decreases because she is better placed to advocate for her rights and preferences. However, when her autonomy reaches a high level, her partner seeks to exercise his dominance and control over her and her resources via emotional IPV.

Strong social connectedness is also found to be relevant for emotional IPV. Although our results differ slightly from those of Mavisakalyan and Rammohan (2021) and Yilmaz (2018), we could nevertheless argue that for a woman at a certain level of IPV risk, as she increases her social interactions, the tensions, conflicts, and disputes with her partner initially rise, leading to a greater likelihood of victimization. After a certain level of social support networks is surpassed, the IPV risk decreases to its initial level.

With regard to the distribution of housework among the family members, we could argue that since this factor is a key gender equality indicator (Ferrant et al., 2014), in families with traditional gender roles, the housework is exclusively done by women, and this inequality is also reflected through emotional IPV. By contrast, households in which only men do the housework seem to represent a safer place for women in terms of IPV victimization.

Our findings at the community level differ to some extent from previous results based on UNiTE Working Group (2019) and WHO (2012). Our results support the existence of a nonlinear relationship between the community’s Gini index and IPV risks. This indicates that lower risks of emotional IPV are observed in women living in highly unequal and highly equal communities. Even though the shape of the estimated relationship differs from previous studies (Rashada & Sharaf, 2016), the results are consistent in terms of the relevance of this factor.

Results regarding the effect of the share of economically active women suggest that a greater degree of women’s economic empowerment in the community’s public life, in particular in job market access, could be generating tensions and conflicts in the private sphere. This may exacerbate existing gender inequalities in the context of intimate relationships, thus increasing women’s IPV victimization risks.

At the societal level, the association found between the prevalence of common crimes against men and women’s likelihood of experiencing emotional IPV makes logical sense. This finding is consistent with previous studies (WHO, 2012).

## Conclusions

In this paper, we aimed to identify the risk factors for emotional IPV against women and girls with children in Mexico. Our theoretical framework is the ecological model, which considers IPV as the result of the interaction and convergence of multiple social, demographic, economic, political, and cultural factors at four interrelated levels: individual, relationship, community, and society. To properly apply the ecological approach and account for the complexity of IPV in our analysis, we integrate a dataset containing 35,004 observations and 42 covariates with information from 10 official sources. Information from the ENDIREH allows us to characterize the individual and relationship levels. Data from the other nine sources (including surveys, censuses, and administrative records) is used to describe the community and society in which the IPV occurs.

The main results confirm the importance of incorporating factors at the four levels of the ecological model, rather than restricting the analysis to only the individual and relationship levels, as done in most previous research. Moreover, we find evidence of linear, nonlinear, and interaction effects describing the links between the analyzed factors and emotional IPV.

At the individual level, we find that young women and/or those who had their first sexual intercourse during childhood face a higher risk of suffering from emotional IPV. At the relationship level, women who marry (or move in together with a partner) late in life, who have a low or a high level of autonomy, who perceive a medium level of support from social networks, and/or who live in a household in which women do all or part of the housework have a higher likelihood of emotional IPV victimization. Protective factors related to community characteristics are high-income inequality or high-income equality and/or a low level of women’s economic participation. A high prevalence of common crimes against men is associated with higher IPV victimization risks at the societal level.

These findings not only yield evidence of risk factors that were either hitherto unknown for the case of Mexico or were based purely on theory without having been tested in empirical studies, but they are also relevant for public policies. In this respect, four key contributions are made by this paper. First, by examining the factors at the individual and relationship levels, we were able to identify some specific risk subgroups of the women population that are generally overlooked; namely, those who had their first sexual intercourse during childhood and women who got married (or moved in together with a partner) late in life. Strategies against IPV should focus on these at-risk groups.

Second, the results about women’s autonomy and social support networks indicate that interventions aiming to promote women’s social and economic empowerment should be accompanied by specific measures to protect women from violence.

Third, even if public policies already seek to promote income equality and women’s economic participation in the community, our findings suggest that these policies should incorporate a gender component regarding IPV, with a particular focus on communities that have a Gini index of around 0.4 and in which a large share of women are economically active.

Finally, anti-crime policies in regions with a high incidence should include programs that also seek to reduce the risk of emotional abuse occurring in the context of intimate relationships.

We leave for further research the application of the proposed methodology to analyze other types of IPV, other years for the case of Mexico, and data from other countries. This will serve to prove the external validity of the results shown in this paper.

## Supplemental Material

sj-pdf-1-jiv-10.1177_08862605211072179 – Supplemental Material for Determinants of Emotional Intimate Partner Violence against Women and Girls with Children in Mexican Households: An Ecological Framework Click here for additional data file. Supplemental Material, sj-pdf-1-jiv-10.1177_08862605211072179 for Determinants of Emotional Intimate Partner Violence against Women and Girls with Children in Mexican Households: An Ecological Framework by Juan A Torres Munguía and Inmaculada Martínez-Zarzoso in Journal of Interpersonal Violence

## Notes

1. Figures show smoothed mean effects with 95% empirical bootstrap confidence intervals. Coefficients express the effect on women’s probability of being victims of IPV and are obtained by using the cumulative standard normal distribution centered on the mean.
