# Supplementary material for: Determinants of Emotional Intimate Partner Violence against Women and Girls with Children in Mexican Households: An Ecological Framework
Source: J Interpers Violence. 2022 Feb 8;37(23-24):NP22704–31. doi: 10.1177/08862605211072179 (PMC9679571; doi:10.1177/08862605211072179)
Supplement: sj-pdf-1-jiv-10.1177_08862605211072179 – Supplemental Material for Determinants of Emotional Intimate Partner Violence against Women and Girls with Children in Mexican Households: An Ecological Framework [file sj-pdf-1-jiv-10.1177_08862605211072179.pdf]

Appendix A. Data integration process.

The process of integrating the ENDIREH with the other nine data sources consists of three steps:

1. From the ENDIREH microdata we select the information related to the questionnaire applied to married or cohabitation women. The observations in these microdata correspond to individual answers given by the respondents to the ENDIREH questionnaire, and each of these individual answers contains a variable to uniquely identify the municipality (CVE_MUN) and the state (CVE_ENT) where the respondent lives. These unique identifiers are assigned by the INEGI (INEGI, 2016).
2. Estimations at the municipal level from the Intercensal Population Survey, CONAPO, UNDP, CONEVAL, homicide records, CNGMD, and the geographic information also contain the municipality unique identifier assigned by INEGI, CVE_MUN. Using this CVE_MUN as a common variable among the datasets, we first merge all the data at the municipal level from these sources, before merging them with the ENDIREH microdata. This results in a database with a two-dimensional tree-like hierarchical structure, in which the individual observations of the ENDIREH microdata (first dimension) are connected to the estimations at the municipal level (second dimension).
3. Finally, the estimations at the state level from the ENCIG and the ENVIPE, which contain the state unique identifier assigned by INEGI, CVE_ENT, are merged with the database resulting from step 2. This results in a database with a three-dimensional tree-like hierarchical structure, *i.e.*, the ENDIREH individual observations (first dimension) are connected to the information at the municipal

level (second dimension), and these, in turn, to the state level estimations (third dimension).

The dataset integration was implemented in R. The corresponding code to replicate this process can be provided on request to the authors.

Appendix B. Data preparation process.

After merging the data sources and identifying the available relevant variables, we carry out the following analysis for each of the covariates:

1. Plausibility. This process consists of inspecting the data to discover potential incorrect coding or data errors, particularly in new covariates derived from existing variables. The three situations analyzed are:
    a. Women's age at first sexual intercourse cannot be greater than women's age at the time of being surveyed.
    b. Women's age at first marriage (or at cohabitation) cannot be greater than women's age at the time of being surveyed.
    c. Women's age at first childbirth cannot be greater than women's age at the time of being surveyed.

    No implausible values were found.
2. Outlier detection. To prevent a few unusual observations from influencing the results, we identify the extreme values and exclude them from the final data. To do this, we create boxplots for the continuous variables.
3. To ensure we have only complete cases in our dataset, we delete all the observations with at least one missing value in one of the covariates used.

The data preparation process was implemented in R. The corresponding code to replicate this process can be provided on request to the authors.

Appendix C. Summary statistics of the final dataset.

Table A1. Summary statistics of categorical variables in the model

Level	Variable	Category	Number of observations	Percentage
Individual	Indigenous origin of the woman	yes	10703	31%
		no	24301	69%
	Formal education level of the woman	low	12591	36%
		medium	20160	58%
		high	2253	6%
	Consent to first sexual intercourse	yes	34310	98%
		no	694	2%
	Pro-gender equality attitude	low	1698	5%
		medium	15809	45%
		high	17497	50%
Relationship	Consent to marriage or cohabitation	yes	32807	94%
		no	2197	6%
	Woman's level of autonomy within the relationship to make decisions about her sexual life	low	1646	5%
		medium	29986	86%
		high	3372	10%
	Woman's level of autonomy within the relationship to make decisions about her professional life and use of economic resources	low	1630	5%
		medium	11582	33%
		high	21792	62%
	Woman's level of autonomy within the relationship to make decisions about her participation in social and political activities	low	1514	4%
		medium	13645	39%
		high	19845	57%
	Division of housework among household members	only males	4895	14%
		both	7417	21%
		only females	22692	65%
	Woman's perception of support from social networks	low	467	1%
		medium	4217	12%
		high	30320	87%
	Level of social interaction reported by the woman	low	7860	22%
		medium	25074	72%
		high	2067	6%
Community	Level of social marginalization	very low	17930	51%
		low	7031	20%
		medium	4811	14%
		high	4305	12%
		very high	927	3%
	Type of community	rural	241	1%
		low urban	2765	8%
		medium urban	12205	35%
		high urban	19793	57%

Table A2. Summary statistics of continuous variables in the model

Level	Variable	standard deviation	min	0.25 quartile	median	mean	0.75 quartile	max
Individual	Age of the woman in years	14.15	16	30	38	40.43	50	80
	Woman's reported monthly earned income, in Mexican Pesos	1372.7	0	0	0	669.69	200	6000
	Age of the woman at first childbirth	3.45	13	17	19	19.85	22	30
	Age of the woman at first sexual intercourse	3.16	10	16	18	18.15	20	28
Relationship	Age of the husband or partner in years	14.62	15	32	41	43.79	54	82
	Husband's or partner's reported monthly earned income, in Mexican Pesos	2735.4	0	1200	3200	3371.4	4800	12000
	Age of the woman at marriage or cohabitation	3.75	12	17	19	19.56	22	33
	Average number of household members per room in the dwelling	1.06	0.4	1.67	2	2.45	3	5
Community	Male share of recent migrant population	0.02	0	0.02	0	0	0.04	0.26
	Human development index	0.07	0.42	0.66	0.7	0.7	0.75	0.94
	Gini index	0.03	0	0.38	0.4	0.4	0.42	0.58
	Economically active men population	8.14	18.32	61.59	66.8	64.9	70.14	83.8
	Men homicide rate per 100,000 men in the municipality	207.76	0	55.67	112.7	176.3	214.97	2392.3
	Total homicide rate per 100,000 people in the municipality	110.13	0	33	62.0	96.7	117.76	1142.4
	Municipal functional capacities index	0.15	0	0.17	0.3	0.3	0.34	0.86
	Share of senior positions in the Municipal Public Administration held by women	0.12	0	0.15	0.2	0.2	0.31	0.88
	Economically active women population	9.97	2.51	17.39	24.49	24.74	32.03	52.25
	Women homicide rate per 100,000 women in the Municipality	24.25	0	0	14.4	20.1	27.73	343.21
	Female share of recent migrant population	0.02	0	0.01	0.02	0.03	0.03	0.26
	Share of the population living in women-headed households	0.05	0.06	0.19	0.2	0.2	0.27	0.39
Region	Share of the population who considered corruption a common or very common problem in their region	0.05	0.75	0.84	0.89	0.87	0.91	0.95
	Share of the population satisfied with the basic public services in their region	0.09	0.24	0.33	0	0.39	0.46	0.54
	Share of common crimes against men not reported to or not registered by the authorities	2.21	87.61	91.29	92.23	92.31	94	96.86
	Prevalence rate of common crimes against men per 100,000 men	7704.3	16477	21391	24318	26204	30077	51555
	Share of common crimes against women not reported to or not registered by the	2.92	88	89.89	92.25	92	94.16	98.06

authorities							
Prevalence rate of common crimes against women per 100,000 women	6293.4	12389	19973	22350	23636	27698	40653

Notes: The summary statistics were calculated in R. The corresponding code to replicate this process can be provided on request to the authors.

Appendix D. Modelling design.

We apply an additive probit regression model to identify the correlates of the women's likelihood of emotional IPV victimization. Formally, let the variable y_i, following a $Bernoulli(\pi_i)$ distribution with probability $\pi_i \in [0,1]$, indicate whether or not the woman i, suffered $(1 = True)$ from emotional IPV during the previous 12 months (between October 2015 and October 2016), for $i = 1, \dots, 35004$ observations. Consider the vectors $\mathbf{w}_{\boldsymbol{i}} \coloneqq (1, \boldsymbol{w}_{i1}, \dots, \boldsymbol{w}_{ip})'$ and $\mathbf{z}_{\boldsymbol{i}} \coloneqq (\boldsymbol{z}_{i1}, \dots, \boldsymbol{z}_{iq})'$ of p categorical and q continuous covariates. Then, the binomial model is given by

$$\eta_i = g^{-1}(\pi_i) = \mathbf{w}_{\boldsymbol{i}}{}'\boldsymbol{\beta} + \sum\nolimits_{k=1}^{q} f_k(\mathbf{z}_{ik}) + \varepsilon_i \qquad (1)$$

For $g(\eta_i) = \pi_i \in [0,1]$, the standard normal cumulative distribution is used. ε_i are the standard normal errors. Model (1) corresponds to a generalized additive model as proposed by Friedman et al. (2000) and Hastie & Tibshirani (1999). Introducing covariate effects from Table 2 into Model (1), the model can be formulated as:

$$\begin{aligned}\eta_i = \boldsymbol{\beta}_0 + \sum\nolimits_{j=1}^{13} \mathbf{w}'_{\boldsymbol{ij}} \boldsymbol{\beta}_j + \sum\nolimits_{k=1}^{26} s_k(\mathbf{z}_{ik}) + \sum_{l=1}^{4} \delta_l(\boldsymbol{interaction_l}) \\ + \sum_{m=1}^{5} \theta_m(\boldsymbol{interaction_m}) + \sum_{s=1}^{2} \vartheta_s(\boldsymbol{rn_s}) + \varphi(\boldsymbol{sp_i}) + \varepsilon_i\end{aligned} \qquad (2)$$

where $\boldsymbol{\beta}_0$ is the model intercept. The rest of the structure of Model (2) can be divided in the following six components:

1. $\sum_{j=1}^{13} \mathbf{w}'_{\boldsymbol{ij}} \boldsymbol{\beta}_j$ represent the parametric component for linear effects of the categorical covariates included in Table 2.

2. $\sum_{k=1}^{26} s_k(\mathbf{z}_{ik})$ is the model component for the effect of the univariate continuous covariates from Table 2. Parameters $s_k(\mathbf{z}_{ik})$ are smoothing functions and all continuous covariates are zero-centered for convergence reasons (Hofner et al., 2014). Given that no specific functional form is established *a priori* for continuous covariates, every function $s_k(\mathbf{z}_{ik})$ is decomposed into an unpenalized polynomial, $\alpha_0 + \alpha_1 \mathbf{z}_{ik}$, and a smooth deviation from this polynomial, $s_k^{centered}(\mathbf{z}_{ik})$. Every $s_k^{centered}(\mathbf{z}_{ik})$ is a smooth P-spline with a second-order difference penalty and 20 equidistant inner knots (Hastie & Tibshirani, 1999; Hofner et al., 2014). Due to the decomposition of $s_k(\mathbf{z}_{ik})$, it can encompass four possible results: non-significant effect, linear effect, non-linear effect, or a combination of linear and nonlinear effects.
3. Interaction effects between a continuous and a categorical variable are denoted by $\sum_{l=1}^{4} \delta_l(\boldsymbol{interaction_l})$. Our aim with these interactions is to estimate age-varying effects on IPV of the categorical variables indigenous origin, education level, consent to first sexual intercourse and consent to marriage (or cohabitation with partner).
4. Component $\sum_{m=1}^{5} \theta_m(\boldsymbol{interaction_m})$ captures the interaction between two continuous covariates, modeled as bivariate P-spline base-learners (Hofner et al., 2014; Hothorn et al., 2020). Our aim with these interactions is to estimate the following effects: age of the woman by age at first childbirth, age of the woman at her first sexual intercourse by the condition of consent, age of the woman by age at her first sexual intercourse, age in years of the woman at marriage or at cohabitation by the condition of consent, age of the woman by age at marriage or at cohabitation,

age of the woman by age of the husband or partner, and woman's reported monthly earned income by husband's or partner's reported monthly earned income.

5. Functions $\sum_{s=1}^{2} \vartheta_{s\tau}(\boldsymbol{rn_s})$ represent random effects capturing the unobserved heterogeneity across municipalities and states, respectively.
6. Geospatial effects are introduced in $\varphi_{\tau}(\boldsymbol{sp_i})$, and are estimated by bivariate tensor product P-splines (Hofner et al., 2014).

The modelling structure was designed in the R package "mboost" (Hothorn et al., 2020). The corresponding code to replicate this process can be provided on request to the authors.

Appendix E. Estimation strategy.

First, we apply the boosting algorithm to estimate the model. This method is a computer-intensive iterative process that combines estimation with automatic identification of significant covariates (variable selection) and determination of the functional form of their linkage with the dependent variable, *i.e.*, model choice (Friedman, 2001). For each of the models estimated in this paper, 5000 initial boosting iterations are performed. Cross-validation is used to prevent overfitting resulting from running this algorithm until convergence and for finding the finite number of iterations, optimizing the prediction accuracy. By doing so, multicollinearity problems are avoided (Hofner et al., 2014).

Second, once the model is fitted at the optimal number of iterations, complementary pairs stability selection with per family error rate control is applied to avoid falsely selecting covariates. By using subsampling procedures, this method simulates a finite number of random subsets of the data, and then, in each of these subsets, it controls the error rate for the number of falsely selected noise variables while selecting relevant variables in the fitting process of the boosting algorithm. After this finite number of subsets have been fitted, the relative selection frequency per covariate effect is determined by calculating the proportion of subsets for which an effect is selected as relevant. All the effects with a relative selection frequency equal to or greater than a previously specified threshold are declared stable effects. For this paper, we set a cutoff of 0.8, *i.e.*, for an effect to be considered stable, it must be selected in at least 80% of the fitted models. As shown in Meinshausen & Bühlmann (2010) results with a cutoff of between 0.6 and 0.9 do not significantly vary. Given the number of potential predictors and their alternative effects in our model, the cutoff of 0.8 corresponds to a per family error rate with a significance level of 0.0398. See Shah & Samworth (2013) for details.

Lastly, 95% confidence intervals for the subset of effects selected as stable are calculated by drawing 1000 random samples from the empirical distribution of the data using a bootstrap approach based on pointwise quantiles (Hofner et al., 2014).

All computations are implemented in the R package “mboost” (Hothorn et al., 2020). The corresponding code to replicate this process can be provided on request to the authors.

Appendix F. Results.

Table A3. Full table of estimation results.

Level	Variable	Categories	Coefficient [95% CI])
Individual	Woman's age		**Linear, slope: -0.003 (Figure 1.a)**
	Indigenous origin	no*	
		yes	
	Formal education level	low*	
		medium	
		high	
	Woman's age by indigenous origin	no	
		yes	
	Woman's age by education level	low	
		medium	
		high	
	Woman's monthly income		
	Woman's age at first childbirth		
	Woman's age by age at first childbirth		
	Woman's age at first sexual intercourse		
	Consent to first sexual intercourse	no*	
		yes	
	Woman's age at first sexual intercourse by condition of consent	no	**Linear, slope: -0.012 (Figure 1.b)**
		yes	**Linear, slope: -0.018 (Figure 1.b)**
	Woman's age by age at first sexual intercourse		
	Pro-gender equality attitude	low*	
		medium	
		high	
Relationship	Partner's age		
	Partner's monthly income		
	Woman's age at marriage with current husband or at cohabitation with current partner		
	Consent to marriage with current husband or to cohabitation with current partner	no*	
		yes	
	Woman's age at marriage with current husband or at cohabitation with current partner by condition of consent	no	
		yes	**Linear, slope: 0.003 (Figure 2)**
	Woman's age by age at marriage or cohabitation		
	Woman's age by partner's age		
	Woman's autonomy within the relationship to make decisions about her sexual life	low*	
		medium	
		high	
	Woman's autonomy within the relationship to make decisions about her professional life and use of economic resources	low*	
		medium	**- 0.1 [-0.129, -0.063]**
		high	
	Woman's autonomy within the relationship to make decisions about her participation in social and political activities	low*	
		medium	
		high	

	Woman's monthly income by partner's monthly income		
	Average number of household members per room in the dwelling		
	Division of housework among household members	only women*	
		both	
		only men	**-0.07 [-0.086, -0.054]**
	Social networks	low*	
		medium	**0.079 [0.062, 0.097]**
		high	
	Woman's level of social interaction	low*	
		medium	
		high	
Community	Male share of recent migrant population		
	Social marginalization level	very low*	
		low	
		medium	
		high	
		very high	
	Type of community	rural*	
		low urban	
		medium urban	
		urban	
	Human development index		
	Gini index		**Non-linear, inverted u-shape (Figure 3.a)**
	Economically active men population		
	Total homicide rate		
	Men homicide rate		
	Municipal functional capacities index		
	Share of senior positions in the local public administration held by women		
	Economically active women population		**Linear, slope: 0.002 (Figure 3.b)**
	Women homicide rate		
	Female share of recent migrant population		
	Share of the population living in woman-headed households		
	Random effects (municipality)		
	Spatial effects		
Society	Share of the population who considered corruption a common or very common problem		
	Share of the population satisfied with the basic public services		
	Share of common crimes against men not reported to or not registered by the authorities		
	Prevalence rate of common crimes against men		**Linear, slope: 0.000003 (Figure 4)**
	Share of common crimes against women not reported to or not registered by the authorities		
	Prevalence rate of common crimes against women		
	Random effects (state)		
